# Development of a Human Dihydroorotate Dehydrogenase (hDHODH) Pharma-Similarity Index Approach with Scaffold-Hopping Strategy for the Design of Novel Potential Inhibitors

**DOI:** 10.1371/journal.pone.0087960

**Published:** 2014-02-04

**Authors:** Kuei-Chung Shih, Chi-Ching Lee, Chi-Neu Tsai, Yu-Shan Lin, Chuan-Yi Tang

**Affiliations:** 1 Department of Computer Science, National Tsing Hua University, Hsinchu, Taiwan; 2 Bioinformatics Center, Chang Gung University, Taoyuan, Taiwan; 3 Graduate Institute of Chang-Gung Medical Science, Chang-Gung University, Taoyuan, Taiwan; 4 Department of Computer Science and Information Engineering, Providence University, Taichung, Taiwan; Concordia University Wisconsin, United States of America

## Abstract

Human dihydroorotate dehydrogenase (hDHODH) is a class-2 dihydroorotate dehydrogenase. Because it is extensively used by proliferating cells, its inhibition in autoimmune and inflammatory diseases, cancers, and multiple sclerosis is of substantial clinical importance. In this study, we had two aims. The first was to develop an hDHODH pharma-similarity index approach (PhSIA) using integrated molecular dynamics calculations, pharmacophore hypothesis, and comparative molecular similarity index analysis (CoMSIA) contour information techniques. The approach, for the discovery and design of novel inhibitors, was based on 25 diverse known hDHODH inhibitors. Three statistical methods were used to verify the performance of hDHODH PhSIA. Fischer’s cross-validation test provided a 98% confidence level and the goodness of hit (GH) test score was 0.61. The *q^2^*, *r^2^*, and predictive *r^2^* values were 0.55, 0.97, and 0.92, respectively, for a partial least squares validation method. In our approach, each diverse inhibitor structure could easily be aligned with contour information, and common substructures were unnecessary. For our second aim, we used the proposed approach to design 13 novel hDHODH inhibitors using a scaffold-hopping strategy. Chemical features of the approach were divided into two groups, and the Vitas-M Laboratory fragment was used to create de novo inhibitors. This approach provides a useful tool for the discovery and design of potential inhibitors of hDHODH, and does not require docking analysis; thus, our method can assist medicinal chemists in their efforts to identify novel inhibitors.

## Introduction

Dihydroorotate dehydrogenase (DHODH) is a highly conserved enzyme that is expressed in all organisms. During the fourth step in a reported pyrimidine biosynthesis, the enzyme catalyzes the oxidation of dihydroorotate to orotate, with concomitant reduction of flavin mononucleotide (FMN) to dihydroflavin mononucleotide (FMNH_2_) [1]. Because DHODH is required to ensure proliferating-cell viability [2], inhibitors have been developed to eliminate human DHODH (hDHODH) activity, which is associated with cancers, multiple sclerosis, and autoimmune and inflammatory diseases (see below) [3].

DHODHs are classified according to cellular location [4], [5]. Class-1 DHODHs are cytoplasmic and single-domain enzymes, whereas class-2 DHODHs are membrane-associated and two-domain enzymes [6]. Both classes of DHODHs use FMN to oxidize DHODH [7]. To regenerate FMN, class-1 enzymes use a soluble cofactor, such as NAD^+^ or fumarate, that binds close to FMNH_2_ [8]. Class-2 enzymes use ubiquinone (CoQ) as the oxidant. CoQ binds in a hydrophobic region of the N-terminal domain, which does not contain an FMN-binding site [7], [9]. Because only class-2 DHODHs contain a CoQ-binding site, we can exploit this binding characteristic in the design of inhibitors that select for a specific DHODH class.

The hDHODH protein is a class-2 enzyme containing 396 residues, and is located in the inner mitochondrial membrane [10], [11]. The enzyme has been associated with rheumatoid arthritis, cancer, and multiple sclerosis [12]–[14], and so, inhibitors of hDHODH have been designed to complex with the CoQ-binding site, thereby reducing the enzyme’s activity [15], [16]. Two such inhibitors, brequinar (BRE) and leflunomide (LEF), have proven effective as drugs against various cancers and rheumatoid diseases [17], [18]. However, the administration of these medications is accompanied by multiple side effects [19], [20]. The crystal structures of hDHODH complexed with analogs of BRE and LEF reveal the formation of strong hydrogen bonds between the inhibitors and hDHODH, illustrating why the BRE and LEF are effective inhibitors of the enzyme [21].

We had two research aims for this study. The first was to construct a computational method for designing novel hDHODH inhibitors. Inhibitor analysis frequently involves the use of 3D-QSAR studies. Two main 3D-QSAR methodologies are the pharmacophore hypothesis [22]–[25], and comparative molecular similarity index analysis (CoMSIA) [26]–[29]. In our survey, several QSAR calculation approaches of DHODH were proposed, such as QSAR (Leban *et al*. [30], Ojha *et al*. [31], and Vyas *et al*. [32]), SOMFA (Shun-Lai *et al*. [33]), and CoMFA/CoMSIA (Vyas *et al*. [34]). For example, Vyas *et al.* [34] used a series of aryl carboxylic acid amide derivatives for QSAR or CoMFA/CoMSIA calculation. These previous approaches have a common factor that is need a common sub-structure for inhibitor alignment calculation. This condition will limit these approaches to discover new diverse inhibitor structures. Our computational method integrates molecular dynamics calculations, pharmacophore hypothesis, and CoMSIA contour information techniques in a pharma-similarity index approach (PhSIA). The diverse inhibitor structures can easily be aligned into the hDHODH PhSIA without the need for a common structure. The hDHODH PhSIA was established by 25 different structures of inhibitors, and that is able to screen for diverse compounds in a database. We applied the first hDHODH PhSIA to identify new inhibitors that match the chemical features of the hDHODH ubiquinone binding site. Our second aim was to design novel hDHODH inhibitors, using PhSIA with a scaffold-hopping method. This strategy easily and quickly identified several novel potential hDHODH inhibitors.

Thus, we report the first hDHODH PhSIA for screening, modifying, and optimizing the chemical structures of potential hDHODH inhibitors in 3D space before synthesis. We demonstrate the method by designing several novel potential inhibitors. The proposed PhSIA method provides excellent predictions of bioactivity and 3D space requirements of novel hDHODH inhibitors.

## Results

### Pharma-Similarity Index Approach

**Figure 1:**
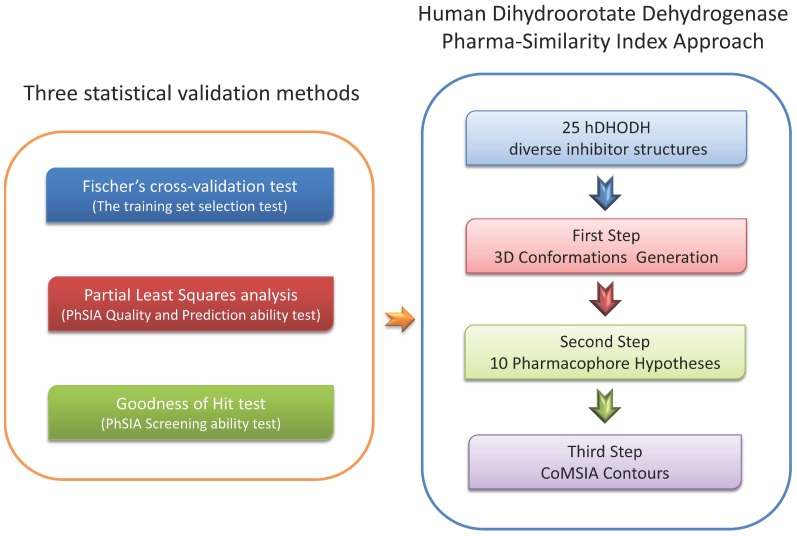
The hDHODH PhSIA generation workflow.

**Figure 2:**
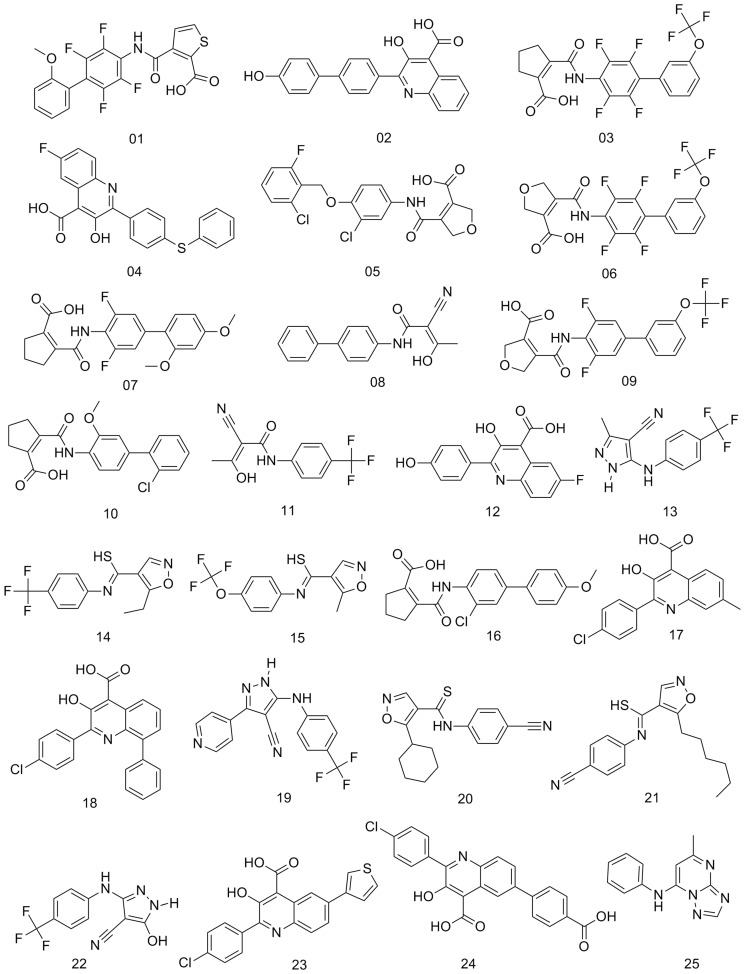
The training set of hDHODH inhibitors used to generate the first hDHODH PhSIA.

Following the approach generation workflow outlined in **Figure 1**, 25 training set inhibitors were selected according to the three rules described in Methods. The structures of training set inhibitors are shown in **Figure 2**. The biological activities of these inhibitors are summarized as pIC_50_ values, shown in **Table 1**. In the second step, ten pharmacophore hypotheses for hDHODH were generated based on the 25 training set inhibitors. Each hypothesis included three hydrogen-bond acceptor (HA) features and one hydrophobic aromatic (HYAR) feature. The fixed and null costs among the ten hypotheses are 83.48 and 399.98 bits, respectively; thus, there is a difference of 316.50 bits between the null and fixed costs. The configuration cost of 9.77 bits is less than the threshold value of 17 bits. Based on these criteria, the total cost of each hypothesis is close to the fixed cost, and is distant from the null cost, indicating that each hypothesis is of high quality. The total cost of the worst hypothesis, Hypo10, is 176.42 bits, and the cost difference of Hypo10 is >70 bits. The correlation coefficient, *r*, of the ten hypotheses ranges from 0.91 to 0.85. **Table 2** summarizes information on the ten pharmacophore hypotheses. A good pharmachphore hypothesis which the *r*-value must larger than 0.9. Compared with the other Hypo models, Hypo01 had the best *r*-value, and thus was the best pharmacophore hypothesis for screening databases and determining the alignment rules for the CoMSIA contour information.

**Table 1:** Actual and Predicted pIC_50_ Values for the Training Set Inhibitors, Based on the Pharma-Similarity Index Approach (PhSIA).

No.	Pharma-Similarity Index Approach	
	Actual pIC_50_	Estimated pIC_50_
1	9	9.07
2	8.22	7.90
3	8.15	8.04
4	7.74	7.97
5	7.39	7.48
6	7.24	7.53
7	7.05	7.04
8	7.05	6.69
9	6.69	6.35
10	6.65	6.71
11	6.38	6.37
12	5.68	5.09
13	5.59	5.31
14	5.52	5.64
15	5.14	5.42
16	5.08	5.55
17	4.77	4.61
18	4.59	4.52
19	4.30	4.24
20	4.30	4.64
21	4.30	4.45
22	4.30	4.27
23	3.87	4.36
24	3.87	3.68
25	3.70	3.62

**Table 2:** The Ten Pharmacophore Hypotheses Generated from the hDHODH Training Set Inhibitors.

Hypothesis no.	Total cost	Cost-diff^a^	Error cost	RMS deviation	Training set (*r*)	Feature^b^
**1**	**142.42**	**256.96**	**126.88**	**2.08**	**0.91**	HA*3, HYAR
2	153.81	246.17	140.70	2.33	0.89	HA*3, HYAR
3	153.93	246.05	139.65	2.32	0.89	HA*3, HYAR
4	154.61	245.37	141.66	2.35	0.89	HA*3, HYAR
5	155.87	244.11	143.71	2.39	0.88	HA*3, HYAR
6	163.84	236.14	152.19	2.52	0.87	HA*3, HYAR
7	164.05	235.93	150.14	2.49	0.87	HA*3, HYAR
8	168.40	231.58	156.60	2.59	0.86	HA*3, HYAR
9	171.43	228.55	159. 90	2.64	0.86	HA*3, HYAR
10	176.42	223.56	164.95	2.72	0.85	HA*3, HYAR

a Cost difference  =  (null cost – total cost), where null cost  =  399.98, fixed cost  =  83.48, and configuration cost  =  9.77. All costs are in units of bits.

b HA, hydrogen bond acceptor; HYAR, hydrophobic aromatic.

Based on these results, we applied the alignment rules to Hypo01 for the generation of CoMSIA contour information in the third step of the approach workflow calculation. The training set for the CoMSIA contour information was the same as that for the pharmacophore training set, and **Table 3** shows the PhSIA results.

**Table 3:** Summary of the PhSIA Analysis.

Parameter	*q^2^*	NC	*r^2^_ncv_*	SEE	*F*-value	*r^2^_pred_*	Contour contributions^a^				
							S	E	H	D	A
PhSIA	0.55	3	0.97	0.28	238.25	0.92	0.18	0.21	0.24	0.24	0.12

a S, steric; E, electrostatic; H, hydrophobic; D, hydrogen bond donor; A, hydrogen bond acceptor.

### Fischer’s Cross-Validation Test

**Figure 3:**
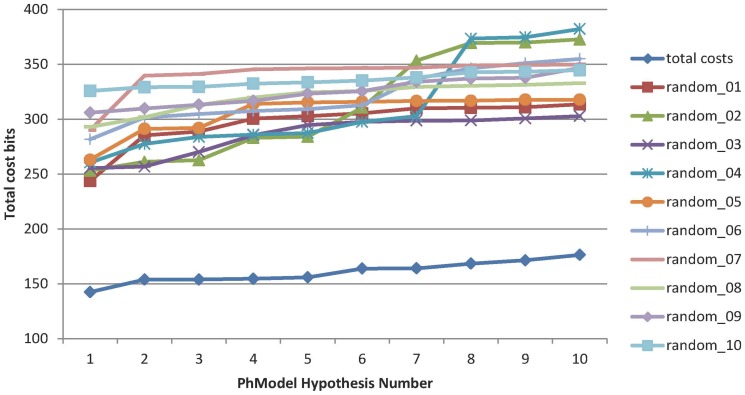
Total costs obtained using the original approach, and for the 10 results that gave the lowest total cost using the 49 randomization procedures approach.

We used Fischer’s test determine the confidence of the training set selection. For this test, set inhibitor pIC_50_ values were randomly assigned to members of the training set to generate 49 random pharmacophore hypotheses; other parameters were used to generate the original set of ten pharmacophore hypotheses. The total costs of the ten original pharmacophore hypotheses was less than that of the 49 random pharmacophore hypotheses obtained after the randomization procedures, indicating a 98% confidence level for the original training set. **Figure 3** summarizes the total costs of the pharmacophore hypotheses and the ten smallest total costs obtained after randomization of the pIC_50_ values, and confirms that the training set could not have been generated from a random selection of inhibitors to generate the PhSIA.

### Partial Least Squares Validation

The testing set of 76 known hDHODH inhibitors was used to validate the PhSIA; the testing set used 76 known hDHODH inhibitors that we did not use for approach generation. The pIC_50_ values of the testing set are shown in **Table S1**. We based our main validation method on the coefficient *r^2^*_pred_, which we used to assess the linearity of relationships between actual and estimated activities. In general, *r* ^2^_pred_ values of >0.5 are considered good.

**Figure 4:**
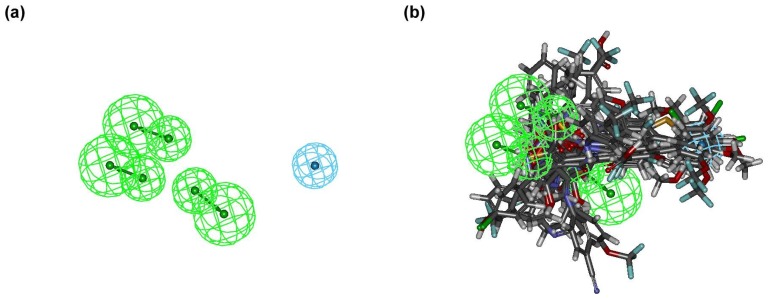
The alignment rule of the hDHODH PhSIA. (a) Chemical features of hypothesis 1 (Hypo01) in approach. (b) The training set of hDHODH inhibitors was aligned based on the chemical features of Hypo01.

**Figure 5:**
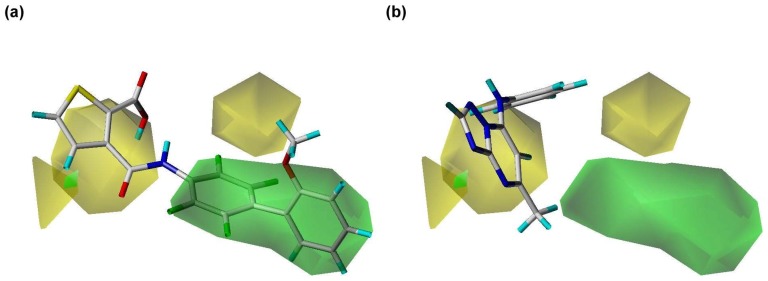
Steric contour regions calculated using the PhSIA. (a) Inhibitor 1 (most active), and (**b**) inhibitor 25 (inactive). Steric allowed and disallowed regions are shown by green (contribution level, 80%) and yellow contours (contribution level, 20%), respectively.

**Figure 6:**
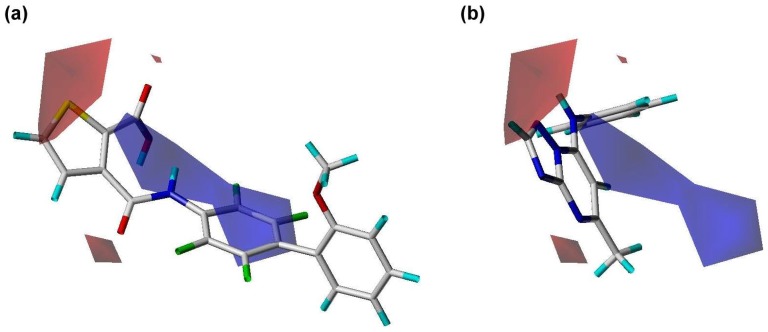
Electrostatic contour regions calculated using the PhSIA. (a) Inhibitor 1 (most active), and (**b**) inhibitor 25 (inactive). Positive charged and negative charged regions are shown by blue (contribution level, 80%) and red contours (contribution level, 20%), respectively.

**Figure 7:**
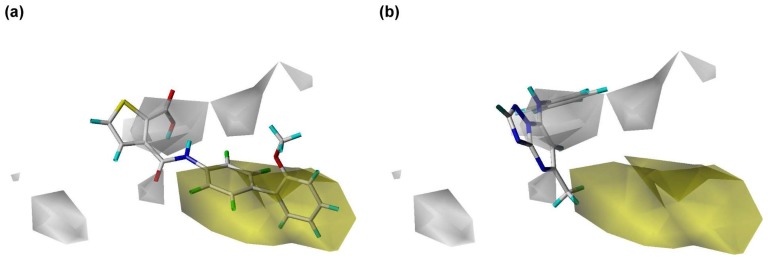
Hydrophobic regions calculated using the PhSIA. (**a**) Inhibitor 1 (most active), and (**b**) inhibitor 25 (inactive). Hydrophobic favored and disfavored regions are shown by yellow contours (contribution level, 80%) and white contours (contribution level, 20%), respectively.

**Figure 8:**
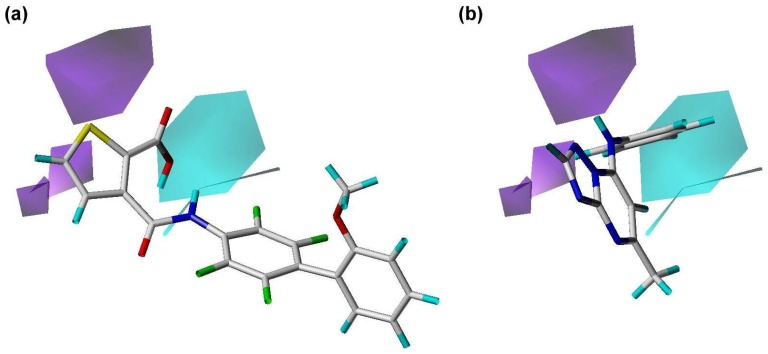
Hydrogen bond donor regions calculated using the PhSIA. (a) Inhibitor 1 (most active), and (**b**) inhibitor 25 (inactive). Hydrogen bond donor favored and disfavored regions are shown by cyan contours (contribution level, 80%) and purple contours (contribution level, 20%), respectively.

**Figure 9:**
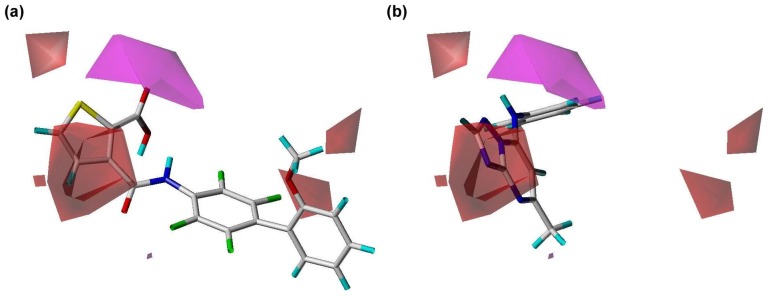
Hydrogen bond acceptor regions calculated using the PhSIA. (a) Inhibitor 1 (most active), and (**b**) inhibitor 25 (inactive). Hydrogen bond acceptor favored and disfavored regions are shown by magenta (contribution level, 80%) and red contours (contribution level, 20%), respectively.

The PhSIA consists of five fields: steric, electrostatic, hydrophobic, hydrogen-bond donor, and hydrogen-bond acceptor. Our approach scored a *q* ^2^ value of 0.55, and the non-cross-validation analysis *r* ^2^ value is 0.97, with a standard error of the estimate (*SEE*) of 0.28 and an F-ratio (*F*) of 238.25. The relative contributions of the steric, electrostatic, hydrophobic, hydrogen-bond donor, and hydrogen-bond acceptor fields are 0.18, 0.21, 0.24, 0.24, and 0.12, respectively (**Table 3** **)**. Estimated pIC_50_ values for the testing set inhibitors were calculated using this approach (**Table S1**). Based on the testing set validation results, the predictive *r* ^2^_pred_ value of the approach is 0.92. The Hypo01 model contains three HA, and one HYAR feature (**Figure 4a**). **Figure 4b** shows the training set aligned onto Hypo01. Contour information for PhSIA is shown in **Figures 5**–**9**.

### Goodness of Hit Test

We used the GH test to assess the ability of our method to identify high-activity inhibitors in the databases. For a good screening level, the value of the GH score must be ≥0.5 [35]. We screened all 229 known hDHODH inhibitors from the ChEMBL database, and 34 inhibitors with high biological activity (pIC_50_ ≥7.3). The results of this analysis are summarized in **Table 4**.

**Table 4:** Statistical Results of GH Test Validation.

Serial no.	Parameter	PhSIA
1	Total no. of inhibitors in database (*D*)	229
2	Total no. of highly active inhibitors in *D* (*A*)	34
3	Predicted no. of highly active inhibitors (*H*_t_)	25
4	Actual no. of highly active inhibitors in *H*_t_ (*H*_a_)	17
5	% Yield of *H*_a_ in *H*_t_ [(*H*_a_/*H*_t_) × 100]	68.0
6	Enrichment factor (*E*) [(*H*_a_ × *D*)/(*H*_t_ × *A*)]	4.58
7	No. of false negatives [*A – H*_a_]	17
8	No. of false positives [*H*_t_ – *H*_a_]	8
9	GH score [Equation 3] a	0.61

a GH test score > 0.6 indicates a very good model.[35].

The PhSIA screening estimated 25 high biological activity inhibitors, of these, 17 actually exhibit high activities, thus, PhSIA scored 68.0% accuracy for the identification of high-activity inhibitors, with a GH score of 0.61. These results show that the PhSIA method offers greater accuracy for database screening.

Thus, our statistical analysis found the hDHODH PhSIA has accurate predictions of inhibition activity, the analysis also confirmed that the chemical features generated by the training set inhibitors are representative, and that the Hypo01 model as good alignment rule for our method.

### Contour Analysis of the Pharma-Similarity Index Approach

The contours generated by PhSIA help provide the 3-dimentional visualization of inhibitors, displaying favored and disfavored regions of the inhibitor structure. Contour information can also facilitate inhibitor optimization, and identify potential analogs. Inhibitor 1 (most activity) and inhibitor 25 (least activity) were used as template structures for contour analysis (**Figures 5**, **6** **,** **7** **,** **8** **and** **9**).

**Figure 5** shows two green and three yellow contours, which denote regions of steric allowed and disallowed space, respectively. Using inhibitor 1 as a template structure, a large steric allowed region is visible as a green contour (**Figure 5a**), and the 1-methoxy-2-methylbenzene group partly inserts into this space. The group’s steric freedom is likely to increase inhibitor activity. For inhibitor 25, there are three regions of steric disallowed space (**Figure 5b**). The inhibitor inserts into the middle yellow contour, accounting for the inhibitor’s limited bioactivity. Thus, the presence of a green contour allows the possibility of increasing the potency of an inhibitor, and the presence of a yellow contour, to decrease the inhibitor’s potency.

The electrostatic contours for positively and negatively charged regions are shown in blue and red, respectively in **Figure 6**. Charged regions are fairly evenly distributed in inhibitor 1 (**Figure 6a**). However, the 3D conformation of inhibitor 25 does not fit the blue and red contours well (**Figure 6b**), possibly accounting for the differences in activity between inhibitors. An inhibitor structure that better fits the electrostatic contours in 3D space, should exhibit increased biological activity.

**Figure 7** clearly shows hydrophobic favored and disfavored regions as yellow and white, respectively. The 2,3,5,6-tetrafluoro-2'-methoxybiphenyl group of inhibitor 1 is surrounded by a hydrophobic favored region, and the propan-2-one group inserts into the disfavored region (**Figure 7a**). The ethane and benzene groups of inhibitor 25 are hydrophobic, however, they are not surrounded by hydrophobic favored regions, but instead are close to disfavored contours (**Figure 7b**). Thus, an inhibitor structure that more closely fits hydrophobic favored regions while avoiding disfavored regions is predicted to exhibit enhanced inhibitory activity.

In **Figure 8**, the hydrogen-bond donor’s favored and disfavored regions are depicted with cyan and purple colored contours, respectively. For inhibitor 1, a methanol group is oriented toward a favored region (**Figure 8a**). In inhibitor 25, the hydrogen-bond donor is a dimethylamine group, which is aligned with a disfavored contour (**Figure 8b**). Additionally, inhibitor 25 is not oriented toward any cyan-colored contours. This illustrates how the various inhibitor structures interact with the hydrogen-bond donor contour, resulting in the observed differenced in hDHODH inhibition activity.

In **Figure 9** shows the contours for the hydrogen-bond acceptor favored and disfavored regions in magenta and red, respectively. The formaldehyde group in inhibitor 1 is aligned with the favored region, and its thiophene moiety inserts into the disfavored region (**Figure 9a**). For inhibitor 25, hydrogen-bond acceptor groups are surrounded by red (disfavored) contours, and no part of the inhibitor oriented toward the magenta (favored) contour (**Figure 9b**); thus, all of the hydrogen-bond acceptor groups in inhibitor 25 align with disfavored regions. This analysis revealed that an inhibitor structure that better fits both the hydrogen-bond acceptor favored and disfavored regions, is predicted to have a greater activity.

Based on the above discussion, contour information describes chemical characteristics and reflects the relative activities of inhibitors. Thus, PhSIA is effective at predicting activity, which could lead to more effective screening, optimization, and modification of designed hDHODH inhibitor structures.

### Design of Novel hDHODH Inhibitors

Based on our analysis, the PhSIA method effectively identifies hDHODH inhibitors, and each inhibitor can be directly screened and analyzed based on contour information. The contour information generated in this approach clearly shows the required chemical features in 3D space, and facilitates optimization of the inhibitor’s molecular weight.

**Figure 10:**
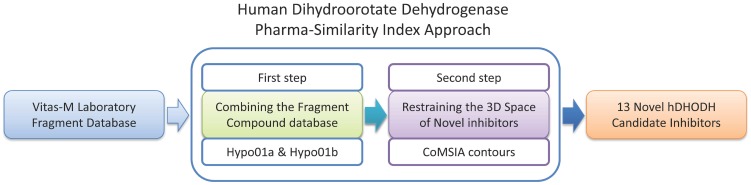
Workflow for the design of novel hDHODH inhibitors.

We applied PhSIA with a scaffold-hopping strategy to design novel hDHODH inhibitors; **Figure 10** shows the design workflow. In the first step, we use Hypo01 to enumerate novel inhibitors. To screen the fragment database, we divide Hypo01 into two parts, Hypo01a and Hypo01b. Hypo01a has three HA features, and Hypo01b has one HYAR feature. To complete the step, Hypo01 generates 13 novel hDHODH inhibitors using the Vitas-M Laboratory fragment database. In the second step, contour information is used to predict biological activity and constrain the molecular weight of the novel hDHODH inhibitors. The estimated biological activities of 13 novel hDHODH inhibitors, their fragments, and structures are shown in **Table S2**.

**Figure 11:**
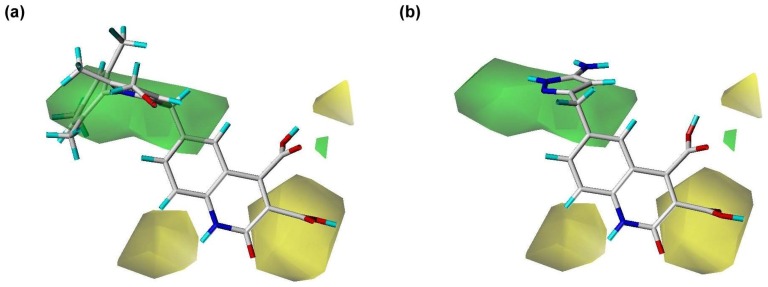
The novel hDHODH inhibitors were constrained by steric contours in PhSIA. **(a)** The novel hDHODH inhibitor (EnFrag01), predicted to highly active. (**b**) The novel hDHODH inhibitor (EnFrag13), predicted to be inactivity.

We used two novel hDHODH inhibitors, EnFrag01 and EnFrag13, with steric contours, as our template for analysis (**Figure 11**). EnFrag01 is predicted a potential hDHODH inhibitor, and is modeled as surrounded by steric contours of allowed and disallowed regions (**Figure 11a**). The EnFrag01 mesitylene group orients to insert into the main green contour, suggesting that the steric tolerance of this chemical moiety would increase inhibitor activity. The 3D EnFrag01 model does not make contact with any steric disallowed regions. EnFrag13, on the other hand, is predicted to be biologically inactive; the main green contour does not surround its 1*H*-pyrazol-5-amine group (**Figure 11b**), indicating that the presence of this bulky group would improve the effectiveness of an inhibitor. Based on contour analysis, the activity of an inhibitor will be greater if the inhibitor’s structure fits well into the tolerated contours.

## Discussion

We successfully developed the first hDHODH PhSIA, based on 25 diverse inhibitor structures, and applied a scaffold-hopping strategy to design 13 novel hDHODH inhibitors. This approach integrated molecular dynamics, pharmacophore hypothesis, and CoMSIA contour information techniques. Three statistical methods were used to validate our approach: Fischer’s test, predictive *r^2^* analysis, and the GH test; the hDHODH PhSIA method identified potential inhibitors and predicted their activity with accuracy. PhSIA can screen inhibitor databases, optimize inhibitor structures, and restrict molecule weight in 3D space, without the need for docking analysis. PhSIA offers several advantages over other methods: (i) the method’s ability to predict biological activity is greater than that using a pharmacophore alone. There are a maximum of five pharmacophore chemical features available as criteria using standard pharmacophore methods. This restriction may result in an incomplete description of the chemical features of an inhibitor, whereas contours generated by CoMSIA do not have this limitation, and thus can more accurately model chemical the features of inhibitor activity, thereby providing better predictions. (ii) The CoMSIA model the restriction that each calculated inhibitor needs a common structure for alignment. This means that diverse inhibitor structures cannot be aligned. In our approach, diverse inhibitor structures can easily be aligned into the CoMSIA models without the need for a common structure. Diverse inhibitors were aligned to the 3D conformations based on the pharmacophore for CoMSIA. Thus, using our approach, the CoMSIA model is able to screen for diverse compounds in a database. (iii) A docking method is generally used after pharmacophore screening to determine the 3D space parameters between an inhibitor and a binding site. In our approach, contour information provides the 3D space boundaries for inhibitor structures. Consequently, it is not necessary to apply docking methods to determine the 3D space boundaries of the binding sites of screened or designed inhibitors. The CoMSIA model provides contours to define the limits of the 3D molecular weight range to fit the target binding site. This approach provides a useful tool for the discovery or design of potential inhibitors of hDHODH, and does not require docking analysis; thus, the method may assist medical chemists in their efforts to identify novel inhibitors.

## Methods

### Biological Data Set

A set of 101 hDHODH inhibitors, collected from the ChEMBL database [36], was used to generate and validate the PhSIA. These inhibitor structures and their biological activities (pIC_50_ values) are shown in **Figure 2** and **Table S1**.

### Constructing the Training and Testing Inhibitor Sets

Constructing the inhibitor training and testing sets is a crucial step for generating and validating the PhSIA. We used the following rules to select the training set inhibitors: (i) a minimum of 16 diverse compounds were selected, to ensure that statistical significance could be properly assessed, (ii) biological activity data associated with the compounds should span at least 3.5 orders of magnitude, and (iii) the training set should include high biological activity and biologically inactive compounds. The remaining inhibitors, not chosen for the training set were assigned to the testing set. Thus, of the 101 inhibitors, 25 were included in the training set (**Figure 2**) and 76 were assigned to the testing set (**Table S1**).

### Generation of the Pharma-Similarity Index Approach

The training set inhibitors were used to generate a PhSIA model, and the testing set of inhibitors was used for validation. The PhSIA model generation workflow comprises three steps (**Figure 1**). The first step is to generate 3D structure conformations of the training set inhibitors. In the second step, these 3D conformations are used to generate ten theoretical pharmacophore hypotheses. In the third step, the “best” pharmacophore hypothesis is determined using specific criteria (described below), and is used as the alignment rule for generation of the CoMSIA contour information.

### Generating the 3D Training set Conformation Structures

The 3D conformations of the training set were generated by molecular dynamics modeling with CHARMm force field parameters [37] using Discovery Studio 2.1 software (Accelrys, Inc., San Diego, CA). The procedure involved the following steps: (i) Conjugate-gradient minimization in torsion space, (ii) Conjugate-gradient minimization in Cartesian space, and (iii) Quasi-Newton minimization in Cartesian space. The conformational-space energy, which corresponds to the maximum energy allowed above the global energy minimum, was constrained to ≤20 kcal/mol. The generated by this stage was kept and performed the structural comparison. If the RMSD (Å) of any of the two snapshots are less than 0.2, compounds were considered as duplicate structure and were removed, and the maximum allowable number of 3D conformations for each inhibitor was restricted to 255.

### Generating the Pharmacophore Hypotheses

The 3D conformations generated in the previous step (Section 4.3.1) were used to produce ten pharmacophore hypotheses using the HypoGen algorithm, as implemented using Discovery Studio 2.1 software. Biological activities of the training set inhibitors are shown in **Table 1**. During the initial phase, the pharmacophore generation procedure considered the HA and HYAR chemical attributes. Default values were used for all other parameters.

A numeric score, called cost was generated during the procedure of pharmacophore hypothesis. The success of each pharmacophore hypothesis was assessed using two important theoretical cost calculations after pharmacophore generation: One was the fixed cost, which represents the simplest model that fits all data perfectly. In the fixed cost hypothesis, the predicted results are exactly the same as the activity values of training set inhibitors. On the contrary, the activity predicted by the null hypothesis is the average activity values of the training set inhibitors. When the difference between the null and the fixed costs is large, the pharmacophore is statistically significant. Specifically, for a “good-quality” pharmacophore hypothesis, the difference between the total cost and null cost is >70 bits, and the total cost should be close to the fixed cost and much lower than the null cost. [22] According to Accelrys Inc. suggestions, the configuration should not be greater than 17.0 in standard HypoGen model. [38]

### Generating the CoMSIA Contour Information

This step was performed using Sybyl-X 1.0 software. Alignment rules were applied to the best pharmacophore hypothesis — the selection of alignment rules is important because they affect the quality of the resulting CoMSIA contour information. Inhibitor 3D conformations were aligned based on the 3D geometric chemical features of the pharmacophore hypothesis. The inhibitor training set inhibitors for establishing CoMSIA contour information were also used for generating the pharmacophore hypothesis.

Gasteiger-Hückel charges were assigned to each of the 3D inhibitor structures. An sp^3^-hybridized carbon atom with a +1 charge was used as the probe for the CoMSIA contour calculation. CHARMm force field parameters were used in the calculation of inhibitor 3D conformations. Five fields, namely steric, electrostatic, hydrophobic, hydrogen-bond acceptor, and hydrogen-bond donor were calculated with an attenuation factor of 0.3 for the CoMSIA contour dataset. A Gaussian distance-dependence function between the probe and each inhibitor atom was applied.

### Validation Methods for the Pharma-Similarity Index Approach

We used three methods to validate the quality of the PhSIA: (i) Fischer’s cross-validation test [23]–[25] (Fischer’s test) was used to assess the confidence of the training set selection; (ii) the partial least squares (PLS) validation method [26]–[29], [39], [40] was used to assess PhSIA prediction quality and accuracy; and (iii) the GH test method [35], [41]–[44] was used to determine the confidence of statistical significance when screening compound databases.

### Fischer’s Cross-Validation Test

We applied Fischer’s randomization test to cross-validate the training set selection. The affinities for the active training set compounds were reshuffled and used to generate pharmacophore hypotheses by taking the same features and parameters used to develop the original set of pharmacophore hypothesis. To achieve a confidence level of 98%, the procedure was performed 49 times, using to the Equation (1), 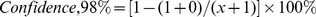
(1)


Where *x* is the number of times the procedure is performed.

### Partial Least Squares Validation Method

PLS analysis of the PhSIA indicated a linear relationship between actual and predicted activities. The filtering column value was set to a default value of 2.0. We also applied the leave-one-out method (described below) to determine the optimal number of components. The *q* ^2^ coefficient was calculated based on a cross-validation analysis. In the leave-one-out method, one inhibitor is removed from the training set, and then, by manipulating the model established by the remaining training set inhibitors, the activity of the removed inhibitor is calculated. When the *r* ^2^_loo_ coefficient is >0.5, we can derive the optimal numbers of components to use in the calculation of *q* ^2^ for the cross-validation analysis, and *r* ^2^ for the non-cross-validation analysis. The predictive term *r* ^2^_pred_ is thus an assessment of the predictive ability of PhSIA, and it is calculated using Equation (2): 
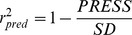
(2)


Where *SD* is the sum of the squared deviations between the actual activity of the testing set and the mean activity of the training set, and *PRESS* is the sum of the squared deviations between the predicted and actual activities for each inhibitor in the testing set.

### Goodness of Hit Test

The GH test was applied to screen the database of known inhibitors to assess the quality of model screening ability in silico. Because the PhSIA can be used to screen databases of hDHODH inhibitors with diverse structures, we applied the GH test to assess the quality of this approach. The GH test score ranges from 0 to 1, where a score of “0” indicates a null approach, and a score of “1” indicates an ideal approach. To calculate the GH test score, we used the following parameters: *D*, the number of known hDHODH inhibitors in the database; *A*, the number of activity inhibitors in the database; *H_t_*, the number of inhibitor hits in the hit list; *H_a_*, the number of activity inhibitors in the hit list; *%Y*, the activity inhibitor percentage yield ; *E*, the enrichment factor; and *GH*, the GH test value. Equation (3) was applied to calculate the GH test score, used to screen databases of known inhibitors:
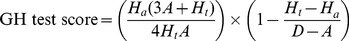
(3)


## Supporting Information

Table S1 **Shows the structures of the testing set inhibitors.** The table provides experimental and estimated pIC_50_ values.
(DOC)

Table S2 **Shows the 13 novel hDHODH inhibitors with their estimated pIC_50_ values.**
(DOC)
