# Supplementary material for: Development of a Human Dihydroorotate Dehydrogenase (hDHODH) Pharma-Similarity Index Approach with Scaffold-Hopping Strategy for the Design of Novel Potential Inhibitors
Source: PLoS One. 2014 Feb 4;9(2):e87960. doi: 10.1371/journal.pone.0087960 (PMC3913663; doi:10.1371/journal.pone.0087960)
Supplement: Table S1 — Shows the structures of the testing set inhibitors. The table provides experimental and estimated pIC50 values. (DOC) [file pone.0087960.s001.doc]

**Supplementary Table S1. The chemical structures of hDHODH testing set inhibitors used for hDHODH Pharma-Similarity Index Approach validations. The experimental and estimated activity pIC_50_ values were based on the Pharma-Similarity Index Approach predictions.**

| No. | Structure | ChEMBL No. | Actual pIC_50_ | Estimated pIC_50_ |
| --- | --- | --- | --- | --- |
| 26 |  | 41719 | 8.30103 | 7.708 |
| 27 |  | 386159 | 8.1549 | 7.831 |
| 28 |  | 218169 | 8.1549 | 7.878 |
| 29 |  | 345701 | 8.09691 | 7.509 |
| 30 |  | 200856 | 8.04576 | 7.803 |
| 31 |  | 219648 | 8 | 7.794 |
| 32 |  | 218249 | 7.92082 | 7.789 |
| 33 |  | 200895 | 7.79588 | 7.308 |
| 34 |  | 484780 | 7.65758 | 7.369 |
| 35 |  | 157105 | 7.48149 | 6.887 |
| 36 |  | 154121 | 7.37675 | 6.887 |
| 37 |  | 371732 | 7.35655 | 6.791 |
| 38 |  | 154513 | 7.34679 | 7.56 |
| 39 |  | 142996 | 7.27572 | 6.89 |
| 40 |  | 157164 | 7.09691 | 6.719 |
| 41 |  | 194776 | 6.97881 | 7.524 |
| 42 |  | 196181 | 6.95861 | 7.155 |
| 43 |  | 197553 | 6.8962 | 7.222 |
| 44 |  | 484531 | 6.88606 | 7.068 |
| 45 |  | 194023 | 6.88273 | 6.884 |
| 46 |  | 155597 | 6.82391 | 6.367 |
| 47 |  | 154372 | 6.76955 | 6.606 |
| 48 |  | 520306 | 6.76955 | 7.317 |
| 49 |  | 195246 | 6.76195 | 6.815 |
| 50 |  | 520008 | 6.72125 | 7.217 |
| 51 |  | 505315 | 6.69897 | 7.122 |
| 52 |  | 483161 | 6.69897 | 6.6 |
| 53 |  | 154493 | 6.55284 | 6.905 |
| 54 |  | 155548 | 6.5376 | 6.679 |
| 55 |  | 381043 | 6.51856 | 6.73 |
| 56 |  | 157277 | 6.50864 | 6.717 |
| 57 |  | 520999 | 6.48149 | 6.994 |
| 58 |  | 154336 | 6.4318 | 6.757 |
| 59 |  | 155530 | 6.40894 | 6.96 |
| 60 |  | 999 | 6.36151 | 6.371 |
| 61 |  | 1088743 | 6.30103 | 6.235 |
| 62 |  | 345685 | 6.21467 | 6.677 |
| 63 |  | 372101 | 6.17588 | 5.701 |
| 64 |  | 194395 | 6.1675 | 6.458 |
| 65 |  | 1088742 | 6.1549 | 6.741 |
| 66 |  | 70544 | 6.09691 | 5.696 |
| 67 |  | 200894 | 6 | 6.443 |
| 68 |  | 426299 | 6 | 6.426 |
| 69 |  | 119801 | 5.49485 | 5.421 |
| 70 |  | 70708 | 5.49485 | 5.077 |
| 71 |  | 120640 | 5.4437 | 5.184 |
| 72 |  | 118468 | 5.39794 | 5.107 |
| 73 |  | 1084927 | 5.25964 | 5.741 |
| 74 |  | 118168 | 5.20761 | 5.782 |
| 75 |  | 418870 | 5.18709 | 5.735 |
| 76 |  | 199574 | 5.07572 | 4.856 |
| 77 |  | 333344 | 5.04576 | 4.983 |
| 78 |  | 305215 | 5.03152 | 5.08 |
| 79 |  | 71154 | 4.95861 | 5.203 |
| 80 |  | 219376 | 4.92082 | 4.723 |
| 81 |  | 200536 | 4.86012 | 4.764 |
| 82 |  | 333404 | 4.7122 | 4.898 |
| 83 |  | 118125 | 4.64975 | 5.11 |
| 84 |  | 218051 | 4.38722 | 4.954 |
| 85 |  | 219520 | 4.36653 | 4.61 |
| 86 |  | 119780 | 4.30103 | 4.856 |
| 87 |  | 118499 | 4.30103 | 4.786 |
| 88 |  | 118455 | 4.30103 | 4.848 |
| 89 |  | 432427 | 4.30103 | 4.509 |
| 90 |  | 492530 | 4 | 4.533 |
| 91 |  | 13380 | 4 | 3.555 |
| 92 |  | 437480 | 4 | 3.482 |
| 93 |  | 452392 | 4 | 3.631 |
| 94 |  | 492720 | 4 | 3.527 |
| 95 |  | 492529 | 4 | 3.526 |
| 96 |  | 452465 | 4 | 3.59 |
| 97 |  | 499296 | 4 | 3.577 |
| 98 |  | 492719 | 4 | 3.656 |
| 99 |  | 492520 | 4 | 3.575 |
| 100 |  | 447995 | 4 | 3.647 |
| 101 |  | 487517 | 3.69897 | 3.793 |
