# Supplementary material for: Development of a Human Dihydroorotate Dehydrogenase (hDHODH) Pharma-Similarity Index Approach with Scaffold-Hopping Strategy for the Design of Novel Potential Inhibitors
Source: PLoS One. 2014 Feb 4;9(2):e87960. doi: 10.1371/journal.pone.0087960 (PMC3913663; doi:10.1371/journal.pone.0087960)
Supplement: Table S2 — Shows the 13 novel hDHODH inhibitors with their estimated pIC50 values. (DOC) [file pone.0087960.s002.doc]

**Supplementary Table S2. The 12 novel hDHODH inhibitors with estimated pIC50 value were generated and predicted based on the hDHODH PhSIA with Vitas-M Laboratory fragment database.**

| **Enumerate Compounds** | | | |  |
| --- | --- | --- | --- | --- |
| **Num** | **Fragment-1** | **Fragment-2** | **Enumerate Fragments** | **PhSIA Estimated pIC_50_** |
| 01 |  |  |  | 7.488 |
| 02 |  |  |  | 7.221 |
| 03 |  |  |  | 7.075 |
| 04 |  |  |  | 7.082 |
| 05 |  |  |  | 7.227 |
| 06 |  |  |  | 7.016 |
| 07 |  |  |  | 7.025 |
| 08 |  |  |  | 7.408 |
| 09 |  |  |  | 7.037 |
| 10 |  |  |  | 7.261 |
| 11 |  |  |  | 7.043 |
| 12 |  |  |  | 7.136 |
| 13 |  |  |  | 5.913 |
